# SARS-CoV-2 infection dysregulates NAD metabolism

**DOI:** 10.3389/fimmu.2023.1158455

**Published:** 2023-06-29

**Authors:** Amin Izadpanah, Joseph C. Mudd, Joe G. N. Garcia, Sudesh Srivastav, Mohamed Abdel-Mohsen, Clovis Palmer, Aaron R. Goldman, Jay K. Kolls, Xuebin Qin, Jay Rappaport

**Affiliations:** ^1^ Tulane National Primate Research Center, Covington, Louisiana, LA, United States; ^2^ Department of Microbiology and Immunology, Tulane University School of Medicine, New Orleans, Louisiana, LA, United States; ^3^ Department of Medicine, College of Medicine Tucson, University of Arizona, Tucson, AZ, United States; ^4^ Biostatistics and Data Science, Tulane University School of Public Health, New Orleans, LA, United States; ^5^ The Wistar Institute, Philadelphia, PA, United States; ^6^ Molecular and Cellular Oncogenesis Program, The Wistar Institute, Philadelphia, PA, United States; ^7^ Proteomics and Metabolomics Shared Resource, The Wistar Institute, Philadelphia, PA, United States; ^8^ Center for Translational Research in Infection and Inflammation, Tulane School of Medicine, New Orleans, Louisiana, LA, United States

**Keywords:** COVID-19, NAD, metabolism, PASC, NAMPT, sirtuins

## Abstract

**Introduction:**

Severe COVID-19 results initially in pulmonary infection and inflammation. Symptoms can persist beyond the period of acute infection, and patients with Post-Acute Sequelae of COVID (PASC) often exhibit a variety of symptoms weeks or months following acute phase resolution including continued pulmonary dysfunction, fatigue, and neurocognitive abnormalities. We hypothesized that dysregulated NAD metabolism contributes to these abnormalities.

**Methods:**

RNAsequencing of lungs from transgenic mice expressing human ACE2 (K18-hACE2) challenged with SARS-CoV-2 revealed upregulation of NAD biosynthetic enzymes, including NAPRT1, NMNAT1, NAMPT, and IDO1 6 days post-infection.

**Results:**

Our data also demonstrate increased gene expression of NAD consuming enzymes: PARP 9,10,14 and CD38. At the same time, SIRT1, a protein deacetylase (requiring NAD as a cofactor and involved in control of inflammation) is downregulated. We confirmed our findings by mining sequencing data from lungs of patients that died from SARS-CoV-2 infection. Our validated findings demonstrating increased NAD turnover in SARS-CoV-2 infection suggested that modulating NAD pathways may alter disease progression and may offer therapeutic benefits. Specifically, we hypothesized that treating K18-hACE2 mice with nicotinamide riboside (NR), a potent NAD precursor, may mitigate lethality and improve recovery from SARS-CoV-2 infection. We also tested the therapeutic potential of an anti- monomeric NAMPT antibody using the same infection model. Treatment with high dose anti-NAMPT antibody resulted in significantly decreased body weight compared to control, which was mitigated by combining HD anti-NAMPT antibody with NR. We observed a significant increase in lipid metabolites, including eicosadienoic acid, oleic acid, and palmitoyl carnitine in the low dose antibody + NR group. We also observed significantly increased nicotinamide related metabolites in NR treated animals.

**Discussion:**

Our data suggest that infection perturbs NAD pathways, identify novel mechanisms that may explain some pathophysiology of CoVID-19 and suggest novel strategies for both treatment and prevention.

## Introduction

Clinical course of COVID-19 can range from asymptomatic to severe pneumonia and life-threatening conditions including acute respiratory distress syndrome (ARDS), shock, and organ failure. Beyond acute SARS-COV-2 pulmonary infection, persistent symptoms develop in patients with Post-Acute Sequelae of COVID (PASC). PASC is characterized by continued pulmonary dysfunction, fatigue, neuropsychiatric, and neurocognitive abnormalities (1, 2).

Metabolic changes contribute to COVID-19 pathophysiology and predisposition to severe outcomes (3, 4). For example, individuals with metabolic syndrome including diabetes, obesity, and hypertension have an increased risk of morbidity and mortality from COVID-19 (5–7). Individuals with advanced age are also more susceptible to severe outcomes and complications from COVID-19 (8). Changes in nicotinamide adenine dinucleotide (NAD) metabolism may play an important role in COVID-19 pathophysiology (9), both in the acute phase and in PASC. Understanding NAD metabolism in the context of COVID-19 is important since NAD is intimately tied with the aging phenotype and several relevant biological pathways, including control of inflammation, tissue regeneration, fibrosis, and oxidative stress. Moreover, deepening our understanding of NAD metabolism during COVID-19 is useful due to the availability of compounds such as nicotinamide riboside (NR) and nicotinamide mononucleotide (NMN) that act as NAD precursors and may hold interventional value.

**Figure 1:**
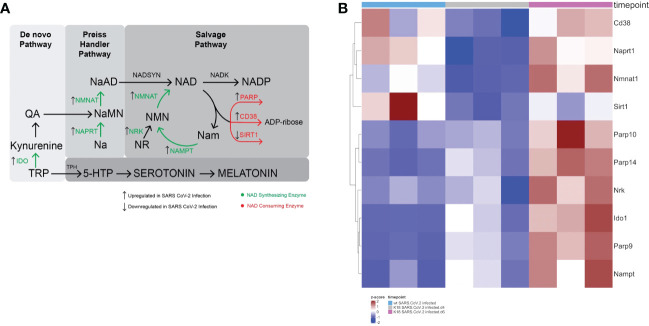
SARS-CoV-2 infection leads to a global perturbation of NAD metabolism. **(A)** NAD anabolic pathways include the *de novo* pathway that begins with tryptophan (TRP), which is metabolized to quinolinic acid (QA). The Preiss Handler pathway begins with nicotinic acid (Na), which is metabolized to nicotinic acid mononucleotide (NaMN) and subsequently to nicotinic acid adenine dinucleotide (NaAD). Consumption of NAD yields nicotinamide (Nam) and ADP-ribose. In the rate limiting step of the NAD salvage pathway, Nam is converted by nicotinamide phosphoribosyltransferase (NAMPT) to nicotinamide mononucleotide (NMN). Nicotinamide mononucleotide adenylyltransferase (NMNAT1) converts NMN to NAD. Nicotinamide inhibits sirtuin and PARP. Nicotinamide may be converted to 1-methylnicotinamide (not shown), which prevents the nicotinamide mediated sirtuin and PARP inhibition. **(B) **Heatmap Demonstrating gene expression changes in NAD synthesizing and consuming enzymes following SARS-CoV-2 infection in K18 hACE2 mice or wildtype mice.

NAD is a coenzyme derived from vitamin B3 (niacin). NAD exists as either an oxidized form NAD+ or a reduced form NADH upon acceptance of hydride, which allows this metabolite to play important roles in reduction-oxidation (redox) reactions. NAD metabolic pathways are detailed in **Figure 1A**. *De novo* synthesis of NAD begins with the amino acid tryptophan, which is an essential amino acid. Indoleamine 2,3-dioxygenase (IDO) converts tryptophan to kynurenine. Kynurenine is further metabolized to quinolinic acid (QA). QA is converted to nicotinic acid mononucleotide (NaMN) by quinolinate phosphoribosyltransferase. The NaMN generated from the *de novo* pathway feeds into the Preiss Handler pathway.

In the Preiss Handler pathway, nicotinic acid phosphribosyltransferase (NAPRT) converts nicotinic acid (Na) to nicotinic acid mononucleotide (NaMN). NaMN is converted to nicotinic acid adenine dinucleotide (NaAD) by nicotinamide mononucleotide adenylyltransferase (NMNAT1-3), which is subsequently converted to NAD by nicotinamide adenine dinucleotide synthetase (NADSYN).

The majority of the cellular NAD pool is replenished by the NAD salvage pathway (10, 11). Consumption of NAD yields nicotinamide (Nam) and ADP-ribose. In the rate limiting step of the NAD salvage pathway, Nam is converted by nicotinamide phosphoribosyltransferase (NAMPT) to nicotinamide mononucleotide (NMN). Nicotinamide mononucleotide adenylyltransferase (NMNAT1) converts NMN to NAD.

NAD functions as a coenzyme or substrate for several reactions. NAD consuming enzymes include poly-ADP ribose polymerases (PARPs), CD38, sirtuins (SIRT), and SARM1. Sirtuins are NAD dependent deacetylases that are present in the mitochondria (SIRT3, SIRT4, and SIRT5), nucleus (SIRT1, SIRT6, and SIRT7), and cytoplasm (SIRT2).

Sirtuins play important roles in the modulation of oxidative stress by deacetylating substrates involved in reactive oxygen species (ROS) generation. For example, SIRT3 deactelyates, and therefore modulates activity of, NUDFA9 (complex I) and SDHA (complex II), which are part of the mitochondrial electron transport chain (ETC) (12–14). SIRT3 is involved in restricting oxidative damage under conditions of caloric restriction (15). SIRT3 has also been shown to counteract ROS through the activation of superoxide dismutase (SOD), which is a ROS scavenger (16). SIRT3 also increases Foxoa3-mediated antioxidant response in the setting of cardiac hypertrophy (17). SIRT1 is also involved in controlling inflammation through the deacetylation of NF-κB (18).

NAD is converted to NADP (nicotinamide adenine dinucleotide phosphate) by NAD kinase. NADPH, which is the reduced form of NADP, is produced by the pentose phosphate pathway. During the conversion of glucose-6-phosphate to 6-phosphogluconate mediated by glucose-6-phosphate dehydrogenase (G6PDH), NADP is reduced to NADPH. NADPH is required for glutathione reductase to convert oxidized glutathione (GSSG) to reduced glutathione (2 GSH). Reduced glutathione is a potent reducing agent which is an important neutralizer of ROS (19).

CD38 (cluster of differentiation 38) is a cyclic ADP ribose hydrolase which consumes NAD to generate ADP-ribose (ADPR) and cyclic ADPR (cADPR), as well as 2-deoxy-ADPR (2dADPR) and nicotinic acid adenine dinucleotide phosphate (NAADP) (20, 21). CD38 has been shown to increase in immune cells and white adipose tissue during aging, and contributes to decreased NAD levels. Cellular senescence, such as that occurs during aging, is associated with decreased proliferation. Despite this, senescence is typically accompanied by a pro-inflammatory senescence-associated secretory phenotype (SASP) involving the secretion of various cytokines including IL-6, IL-8, and IL-1b (22, 23). Notably, CD38 is also upregulated in senescent cells leading to NAD decline (24, 25) and may be a component of SASP. Increased CD38 has been shown to be involved in NAD decline and decreasing mitochondrial function during aging (26).

The involvement of NAD in the interplay of infection, inflammation, and aging led us to hypothesize that SARS-CoV-2 infection induces changes in NAD metabolism that may underly pathophysiology of COVID-19.

## Results

### SARS-CoV-2 infection perturbs NAD metabolism

K18-hACE2 mice, which express human ACE2 in airway epithelium and are therefore susceptible to SARS-CoV-2 infection, were inoculated with SARS-CoV-2 *via* intranasal route. Wildtype C57BL/6 mice, which are not susceptible to SARS-CoV-2 infection due to the lack of human ACE2, served as control (27). We mined the data from our previous published data on bulk RNA analysis of SARS-CoV2-infected K18 lungs as compared with SARS-CoV2-infected B6 mice at at day 4 and day 6 post infection (27)(GSE175996).

We found that there was upregulation of several genes involved in NAD anabolism, including *Naprt1, Nmnat1, Nrk, Ido1, and Nampt* in infected lungs. Simultaneously, genes involved in NAD catabolism were also upregulated, including *Cd38, Parp9, Parp10, and Parp14*. Interestingly, *Sirt1*, an NAD consuming enzyme that plays important roles in control of inflammation due to its deacetylase activity, was downregulated (**Figure 1B**). **Figure 1A** depicts the major NAD synthesizing and catabolizing pathways and the effect of SARS-CoV-2 infection on the RNA levels of pathway enzymes. Altogether, the upregulation of enzymes involved in both NAD anabolism and catabolism suggest that SARS-CoV-2 infection increases NAD turnover.

**Figure 2:**
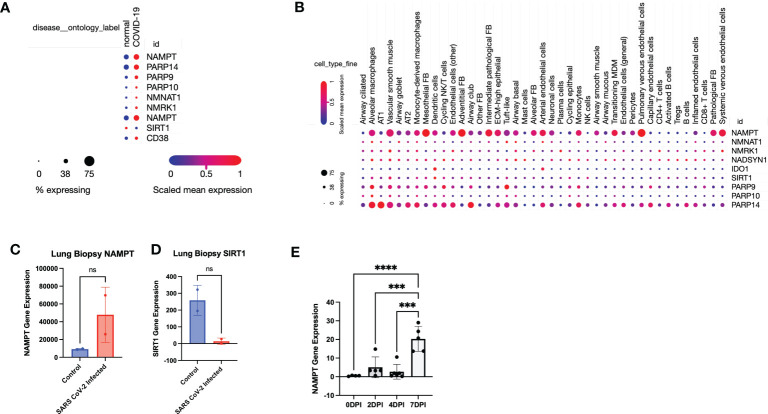
Data mining confirms that SARS-CoV-2 infection leads to a global perturbation of NAD metabolism. **(A)** RNA sequencing from lungs from individuals with COVID-19 versus controls revealed upregulation of NAD anabolic and catabolic enzymes. **(B)** Single Cell stratification of gene expression changes (singlecell.broadinstitute.org) (28)(GSE171524). **(C)** NAMPT and **(D)** SIRT1 levels from SARS-CoV-2 infected lungs versus control (accessed through GSE147507). **(E)** NAMPT levels from SARS-CoV-2 infected K18-hACE2 mice (accessed through GSE154104). ***p<0.001. ns, not significant.

We next validated our findings through further mining published data. Using the established single-cell lung atlas of lethal COVID-19 (28) (GSE171524), we found that SARS-CoV-2 induced gene expression changes in human lungs (**Figure 2A**) that was consistent with our findings in the mouse (**Figure 1**). Despite a global or aggregate upregulation in NAD anabolic and catabolic enzymes, single cell analysis revealed that upregulation of NAMPT was highest in mesothelial fibroblasts, adventitial fibroblasts, and pulmonary venous endothelial cells (**Figure 2B**).

Interestingly, Type 1 alveolar cells (AT1) and Type 2 alveolar cells (AT2) did not have a robust upregulation of NAMPT, despite a higher expression of PARP14, particularly in AT1 cells (**Figure 2B**). This suggests that despite an aggregate increase in NAD synthesizing enzymes, particularly NAMPT, the effect may not be as robust in AT1 and AT2 cells which constitute most of the pulmonary parenchyma. Further data mining reflected an increase in NAMPT RNA levels and decrease in SIRT levels in SARS-CoV-2 infected lungs, compared to non-SARS-CoV-2 infected lungs (29) (**Figures 2C, D**). We also mined data from another group using a similar K18-hACE2 model of SARS-CoV-2 infection and found a time-dependent increase in Nampt levels in lungs (30) (**Figure 2E**) (GSE154104).

**Figure 3:**
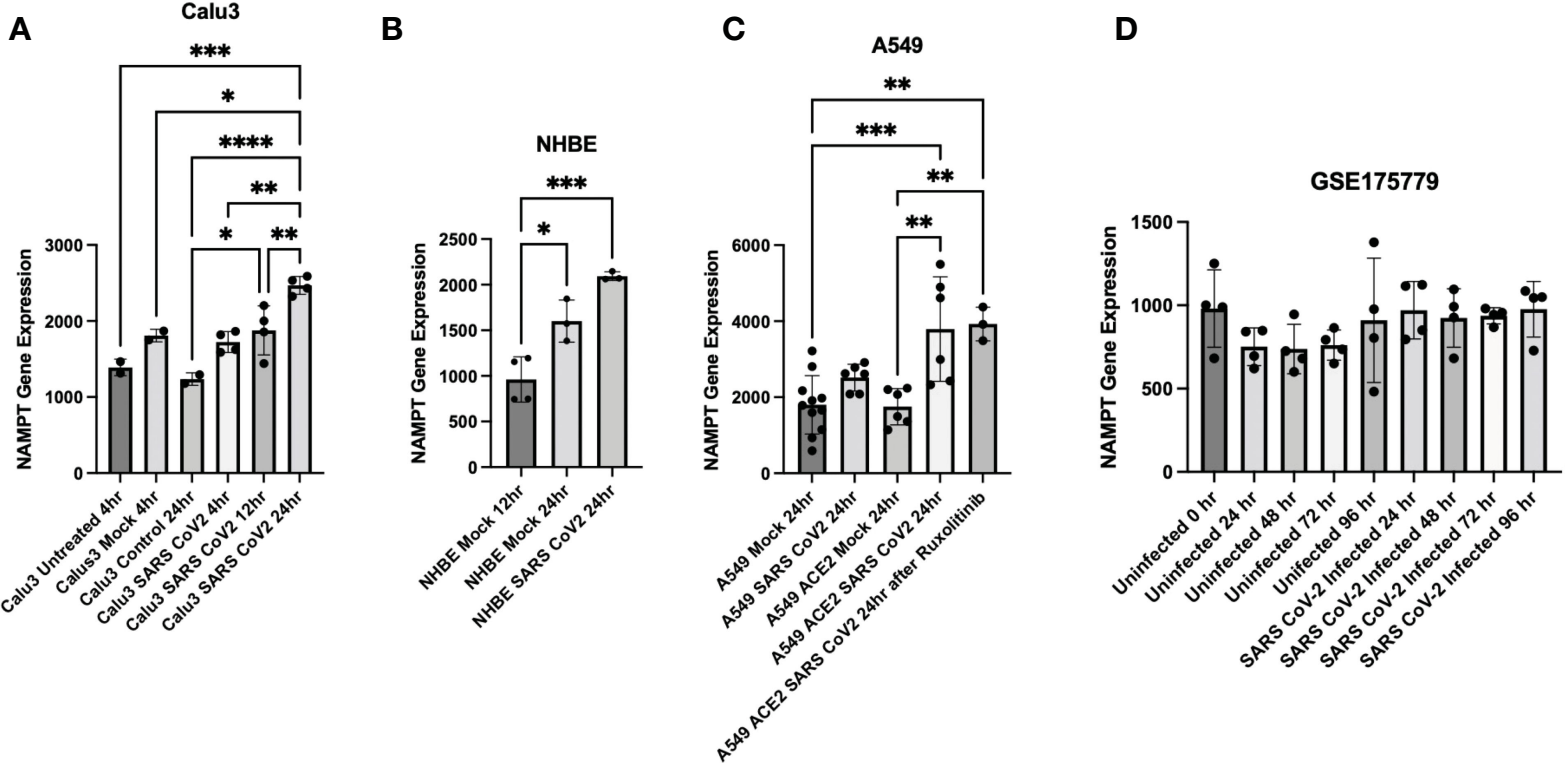
Cell culture experiments demonstrate upregulation of NAMPT in response to SARS-CoV-2 infection. SARS-CoV-2 infection induced NAMPT gene expression in **(A)** Calu3 cells, **(B)** NHBE cells, and **(C)** A549 cells (GSE147507, Data was accessed through Skyline Immgen (www.immgen.org)). **(D)** SARS-CoV-2 infection failed to induce a robust increase in NAMPT in primary human airway epithelial cells from aged individuals (GSE175779). ANOVA was used followed by Tukey’s multiple comparisons test. *p<0.05, **p<0.01, ***p<0.001, ****p<0.0001.

Further data mining found that SARS-CoV-2 infection results in increased NAMPT gene expression in three pulmonary cell lines: Calu3, NHBE, and A549 (data from (29)/GSE147507 and accessed through Skyline Immgen (www.immgen.org) (**Figures 3A**–**C**). Calu3 cells are a submucosal gland cell line generated from a bronchial adenocarcinoma. A549 cells are adenocarcinoma alveolar basal epithelial cells. NHBE cells are normal human bronchial epithelial cells isolated from above the bifurcation of the lungs.

Interestingly, SARS-CoV-2 infection of primary human airway epithelial cells from aged individuals failed to elicit a robust increase in NAMPT (31) (**Figure 3D**). This may suggest that lungs of aged individuals have decreased capacity to upregulate NAMPT as compensation for increased NAD turnover from SARS-CoV-2 infection.

### Effect of NR and Anti-monomeric NAMPT antibody on SARS-CoV-2 infection

Our validated findings demonstrating increased NAD turnover in SARS-CoV-2 infection suggested that modulating NAD pathways may alter disease progression and may offer therapeutic benefits. Specifically, we hypothesized that treating K18-hACE2 mice with nicotinamide riboside (NR), a potent NAD precursor, may mitigate lethality and improve recovery from SARS-CoV-2 infection. We also tested the therapeutic potential of an anti- monomeric NAMPT antibody using the same infection model. Nicotinamide phosphoribosyltransferase (NAMPT) is the rate-limiting biosynthetic enzyme in the salvage NAD synthesis pathway and exists intracellularly (iNAMPT) and extracellularly (eNAMPT) (32). NAMPT can exist in either a monomeric or dimeric configuration (33). Monomeric NAMPT may promote pro-inflammatory effects that contribute to diabetes (34, 35) and ARDS in mouse model (36). While small molecular inhibitors (FK866 and GMX1778) are available, they will block all NAMPT and prevent the ability to dissect the role of monomeric NAMPT. We chose this antibody based on previous report of anti-monomeric NAMPT activity (35). Since data suggests that that SARS-CoV-2 dysregulates NAD metabolism by increasing NAD turnover, boosting NAD levels may promote cellular NAD balance and potentially alleviate the dysregulation. Blockade of monomeric NAMPT would tend to inhibit the NAD-independent effects of NAMPT, and maintain NAD-dependent effects of NAMPT.

**Figure 4:**
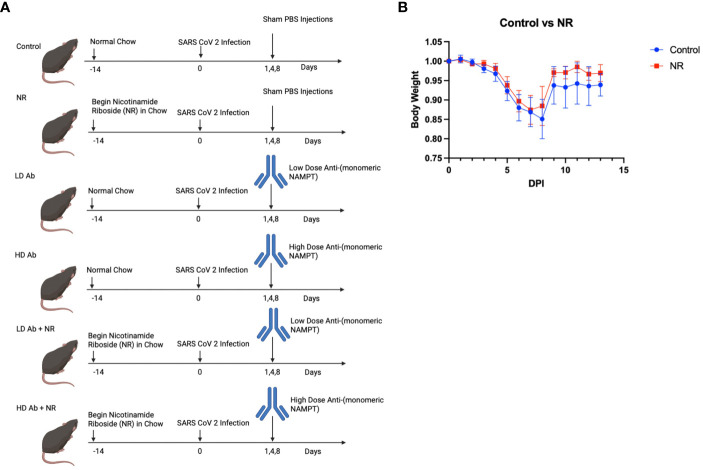
Study design and Effect of NR on SARS-CoV-2 infection. **(A)** Study design shows the time course of the experiment and interventions Created with BioRender.com. **(B)** Body weight curves showing body weight changes for infected control animals versus NR treated animals (n=6). Animals receiving NR had statistically not significant (p>0.05) increased mean body weight, compared to control.

In the study demonstrated in **Figure 4A**, six groups of K18-hACE2 mice (n=6 mice per group) were infected with sublethal dose of SARS-CoV-2 and followed for 14 days post infection. NR was added to the chow for NR receiving groups. Low dose (LD) anti-monomeric NAMPT (Ab) or high dose (HD) Ab was administered at days 1,4, and 8 post infection, either as monotherapy or in combination with NR.

**Figure 5:**
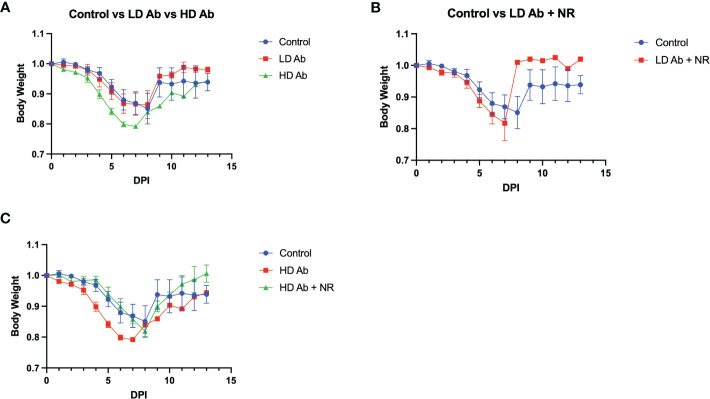
Effect of Anti-monomeric NAMPT antibody on SARS-CoV-2 infection. **(A)** Body weight changes from control animals versus LD Ab treated and HD Ab treated animals. **(B)** Body weight changes from control animals versus LD Ab + NR treated animals. **(C)** Body weight changes from control animals versus HD Ab treated and HD Ab + NR treated animals. The HD Ab treated animals had decreased body weight, compared to control at 2 DPI (*p=0.038*), 4 DPI (*p=0.047*), and 5 DPI (*p=0.047*).

Results indicate that animals receiving NR had statistically not significant increased mean body weight, compared to control, throughout the course of the experiment (**Figure 4B**). The LD Ab treated animals also had increased mean body weight, compared to control, during the recovery phase after 8 days post infection (DPI) (**Figure 5A**). However, the HD Ab treated animals had decreased body weight, compared to control at 2 DPI (*p=0.038*), 4 DPI (*p=0.047*), and 5 DPI (*p=0.047*). This is possibly due to blocking both dimeric and monomeric NAMPT (**Figure 5A**). Combining HD Ab with NR mitigated the rapid weight loss during early infection, suggesting a potential beneficial effect of NR (**Figure 5C**).

**Figure 6:**
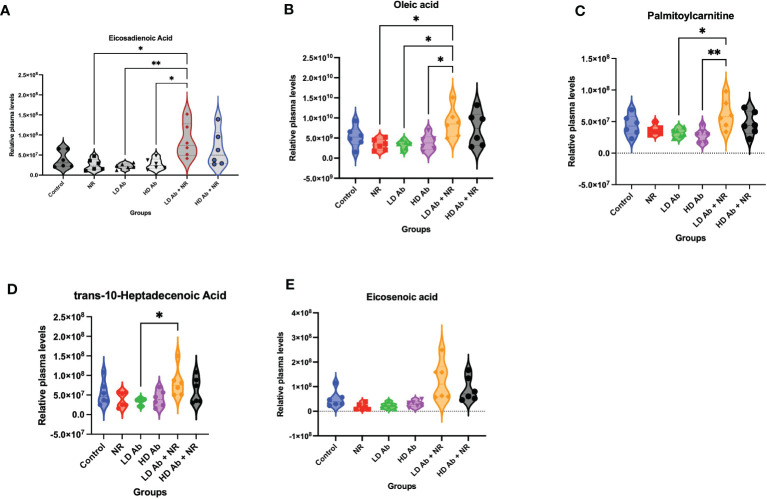
Metabolomic analysis of plasma from study animals. Metabolomic analysis was conducted on plasma collected from study animals. Plasma levels of **(A)** eicosadienoic acid, **(B)** oleic acid, **(C)** palmitoylcarnitine, **(D)** trans-10-heptadecanoic acid, and **(E)** eicosenoic acid are shown. Data was analyzed by ANOVA in GraphPad Prism followed by Tukey’s multiple comparisons test *p<0.05, **p<0.01.

Metabolomic analysis on plasma collected from the K18-hACE2 mice from this study demonstrated a significant increase in lipid metabolites in the LD Ab+NR group which may have anti-inflammatory effects. For example, eicosadienoic acid and oleic acid were significantly increased in LD Ab+NR, compared to NR, LD Ab, and HD Ab groups (**Figures 6A, B**). Palmitoylcarnitine was significantly increased in LD Ab + NR, compared to LD Ab and HD Ab groups (**Figure 6C**). The LD Ab + NR group also showed significantly increased trans-10-heptadecenoic acid, compared to LD Ab alone (**Figure 6D**). Eicosenoic acid levels were increased in LD Ab+NR, compared to NR, LD Ab, and HD Ab groups, but this did not achieve statistical significance (**Figure 6E**).

**Figure 7:**
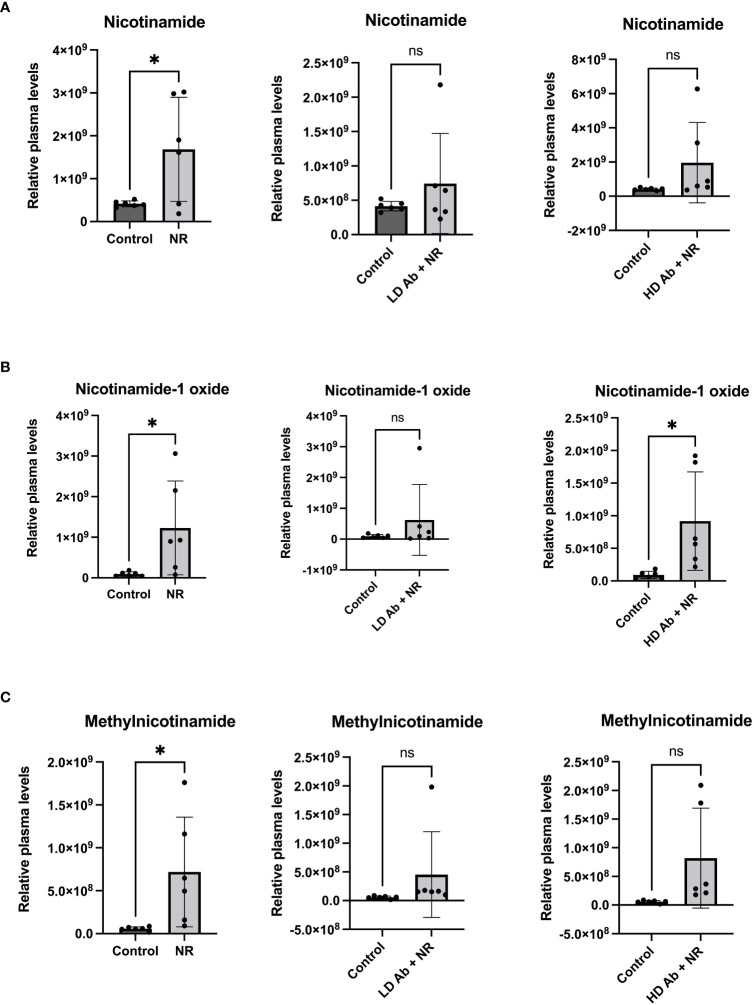
Metabolomic analysis reveals increased nicotinamide metabolites in NR treated animals. Metabolomic analysis was conducted on plasma collected from study animals. Plasma levels of **(A)** nicotinamide, **(B)** nicotinamide-1-oxide, and **(C)** methylnicotinamide are shown. T-test, *p<0.05. ns, not significant.

The NR treated group demonstrated statistically significant increased nicotinamide related metabolites including nicotinamide, nicotinamide-1-oxide, and methylnicotinamide, compared to control (p<0.05, **Figure 7**). The NR + LD Ab and NR + HD Ab groups demonstrated increased nicotinamide, nicotinamide-1-oxide, and methylnicotinamide levels, compared to control, but this did not achieve statistical significance, with the exception of nicotinamide-1-oxide, which was significantly increased in NR + HD Ab compared to control (p<0.05, **Figure 7**).

## Discussion

Our findings demonstrate that SARS-CoV-2 infection increases enzymes involved in both NAD synthesis and degradation, suggesting an increase in NAD turnover. SARS-CoV-2 has been shown to upregulate PARPs and reduce cellular NAD (37). PARPs can be stimulated by interferon signaling (38) and have been shown to have antiviral properties through ADP-ribosylation (39). However, PARPs require NAD for their enzymatic activity and therefore, PARP upregulation can deplete cellular NAD. Increased kynurenine/tryptophan ratio, a reflection of IDO1 activity, has previously been reported during SARS-CoV-2 infection in both non-human primate (40) and humans (41). PARP inhibitors have been suggested as therapeutics for COVID-19 due to their ability to curb inflammation by decreasing Il-6, Il-1, and TNF-a (reviewed in (42)). Nicotinamide has also been suggested as a therapeutic since it inhibits PARPs and serves as a precursor for NAD in the salvage pathway (43). However, nicotinamide is also an inhibitor of sirtuins, which are critical in controlling inflammation (44, 45), and therefore nicotinamide may possibly exacerbate inflammation or delay its resolution. Increased CD38 activity in COVID-19 is thought to contribute to NAD decline and may increase susceptibility to poor outcomes in aged individuals, who have increased baseline CD38 activity due to advanced age (46, 47).

Moreover, in aging, NAD levels decrease (48). Aged individuals may have reduced capability to support healthy NAD levels in response to infection, explaining predisposition of the elderly to severe COVID-19 outcomes. Continued inflammation in the subacute and chronic phases of COVID-19 may compromise ability to maintain NAD balance, leading to deleterious effects. For example, NAD is synthesized *via* a *de novo* pathway initiated by conversion of tryptophan to kynurenine by IDO. Our data demonstrates that SARS-CoV-2 infection upregulates enzymes in the *de novo* NAD synthesis pathway, Preiss Handler pathway, and the salvage pathway (**Figure 1**). Upregulation of IDO may be associated with or even a compensation for NAD deficiency. Kynurenine is metabolized to quinolinic acid, which promotes neurotoxic effects. Tryptophan is also required for synthesis of serotonin and melatonin necessary to stabilize mood and promote sleep. Increased IDO and inflammation could promote bias toward kynurenine synthesis at the expense of the alternative methoxyindole pathway leading to serotonin and melatonin depletion (**Figure 1A**). Since IDO directly catabolizes serotonin, increased IDO activity may lead to mood and sleep disorders (the latter since melatonin is generated from serotonin), which are particularly relevant hallmarks of PASC. Increased IDO activity has also been shown to be a biomarker of inflammation during other viral infections including HIV (49).

Our data suggests that it may be possible that type 1 and type 2 pneumocytes do not upregulate NAMPT to a large extent in response to infection. Type 1 pneumocytes are thin squamous cells that line the alveoli and are involved in gas exchange. Type 2 pneumocytes are cuboidal alveolar cells are principally involved in the production and secretion of surfactant, which reduces surface tension within the alveoli and prevents alveolar collapse (50). Additionally, following lung injury, type 2 pneumocytes act as stem/progenitor cells and self-renew and replace damaged type 1 pneumocytes (50). Acute respiratory distress syndrome (ARDS), which is a notable feature of severe, acute COVID-19, results in damage to both type 1 and type 2 pneumocytes (51). Destruction of type 1 pneumocytes arising from increased inflammatory cytokines and infiltration of immune cells compromises gas exchange. Loss of type 2 pneumocytes results in decreased surfactant secretion and alveolar collapse, leading to intrapulmonary shunting and hypoxemia. In cases of nonlethal disease, loss of type 2 pneumocytes may prevent pulmonary regeneration, and progressive interstitial fibrosis results in response to fibroblastic infiltration and proliferation, which may progress to restrictive lung disease.

Several lines of evidence suggest the role of NAD turnover in the pathophysiology of pulmonary disease and the process of aging. Senescence of type 2 pneumocytes was demonstrated to be mitigated by co-culture with MSCs, which promoted NAMPT and NAD levels in type 2 pneumocytes (52). Consistently, mesenchymal stem cells (MSCs) were shown to attenuate bleomycin induced pulmonary fibrosis in mice (52). Alveolar epithelial cell aging resulting from increased CD38 promoted pulmonary fibrosis; inhibition of CD38 mitigated bleomycin induced fibrosis (53). Elevated CD38 has been reported in systemic sclerosis, a severe fibrosing disease (54). Blockade of CD38 or supplementation with nicotinamide riboside (NR) protects mice from skin, lung, and skin fibrosis in a bleomycin model of fibrosis (54). The pathophysiology is thought to involve inflammatory synthesis of extracellular matrix due to fibroblastic proliferation, collagen deposition, and fibrosis. Signs and symptoms stem reflect underlying fibrosis. Restoring SIRT3 gene expression ameliorated age related pulmonary fibrosis in mice (55). These suggest that failure to increase or maintain NAD anabolism, or excessive increase in NAD catabolism, can generate imbalance in NAD metabolism and promote nonregenerative, fibrotic outcomes.

We observed a significant increase in lipid metabolites, including eicosadienoic acid, oleic acid, and palmitoyl carnitine in the LD Ab + NR group (**Figure 6**). We also observed significantly increased nicotinamide related metabolites in NR treated animals (**Figure 7**). Eicosadienoic acid is an omega-6 fatty acid with inflammation modulating effects (56). Oleic acid is an omega-9 fatty acid with anti-inflammatory effects (57). Oleic acid has been shown to decrease mortality in an experimental sepsis model (58). Deficiency in oleic acid may contribute to gut inflammation (59). Interestingly, gut dysfunction has been recently reported in patients with COVID-19 (60). Oleic acid has also been shown to contribute to neural stem cell survival and hippocampal neurogenesis (61). Further dissection of role of oleic acid in COVID-19 is warranted, especially as the neurological sequelae of COVID-19 becomes elucidated. Oleic acid has also been shown to protect against oxidative stress in fibroblasts (62).

Palmitoylcarnitine is essential in the B-oxidation of long-chain fatty acids (63, 64), and therefore may suggest an effect on fatty acid metabolism. Palmitoylcarnitine has also been shown to play a role in neural differentiation (65). Palmitoylcarnitine reduces protein kinase C (PKC) activity (66). Interestingly, PKC inhibitors have been shown to reduce the replication of SARS-CoV-2 in cell culture (67). Further studies should elucidate metabolic changes during SARS-CoV-2 infection. It is of particular interest to determine whether there is an increased fatty acid metabolism, and how this influences disease progression.

Increases in nicotinamide metabolites suggest modulation of nicotinamide metabolism. Nicotinamide undergoes oxidation in the liver to generate Nicotinamide-1-oxide (also known as Nicotinamide N oxide) for excretion (68). It was previously shown that mice given a high fat diet to induce obesity had increased levels of nicotinamide oxide as well as 1-methyl nicotinamide, suggesting an overall perturbation of NAD metabolism (69). Increases in nicotinamide derivatives may suggest excretion of nicotinamide substrate, which may be suggestive of activated nicotinamide metabolism. This may alter cellular NAD synthesis and levels, since the salvage pathway relies on the recycling of nicotinamide. The increased NAD turnover may result in upregulation of NAD synthesizing pathways, which is consistent with what we observed during SARS-CoV-2 infection. Increases in 1-MNA may reflect depletion of nicotinamide. Since nicotinamide is an inhibitor of sirtuins (44), loss of nicotinamide through generation of 1-MNA may serve as a compensatory mechanism to maintain sirtuin activity. However, it may also be true that loss of nicotinamide results in slowing of the salvage pathway, which relies on recycling nicotinamide into NAD, which is required for sirtuin function. Similarly, nicotinamide is an inhibitor of PARP (70). While PARP upregulation during infection may serve to promote DNA repair from insults, continued PARP upregulation will deplete cellular NAD and result in cellular death. Increases in 1-MNA may relieve nicotinamide mediated PARP inhibition. Therefore, further investigation is required to understand the interplay between nicotinamide metabolites including 1-MNA and sirtuin and PARP activity, particularly in the context of SARS-CoV-2 infection.

These findings suggest that modulation of NAD pathways has affects systemic metabolism in the context of SARS-CoV-2 infection.

## Conclusions and limitations

Our findings suggest that SARS-Cov-2 infection upregulates molecules involved in both NAD synthesis and degradation, suggesting increased NAD turnover. Our data mining results confirm our findings and suggest that during infection, type 1 and type 2 pneumocytes may not compensate for the increased NAD demand by upregulating NAMPT. This may result in exacerbation of ARDS and poor prognosis. We observed a significant increase in lipid metabolites, including eicosadienoic acid, oleic acid, and palmitoyl carnitine in animals treated with LD Ab + NR (**Figure 6**). Notably, HD anti-NAMPT antibody resulted in significantly decreased body weight compared to control, which was mitigated by combining HD anti-NAMPT antibody with NR (**Figure 5**). It may be likely that inhibition of NAMPT decreases supply of NAD despite an increased demand due to infection, which can be alleviated by NR, an NAD precursor. We observed that animals treated with NR had consistently increased mean body weights compared to control during the recovery phase of the infection (**Figure 4**). We also observed significantly increased nicotinamide related metabolites in NR treated animals (**Figure 7**). Despite these important findings, our study was underpowered to demonstrate a statistically significant benefit for NR in increasing body weight, compared to control. Increased sample size would likely lead to statistically significant results. Nevertheless, our studies and data mining results demonstrate a clear involvement of NAD related pathways, metabolites, and enzymes in SARS-CoV-2 pathophysiology. Future directions will further dissect the effect of modulation of NAD levels and NAMPT levels and activity on SARS-CoV-2 infection.

## Methods

### K18-hACE2 SARS-CoV-2 infection model

Animals were obtained from Jackson Laboratories. Animals were housed in the Tulane University Department of Comparative Medicine. All animal studies were approved by Tulane University Institutional Animal Care and Use Committee (IACUC).

K18-hACE2 (034860) mice from the Jackson Laboratory, which express human ACE2 and are there susceptible to SARS-CoV-2 infection, were inoculated with SARS-CoV-2 *via* intranasal route. Wildtype C57BL/6 mice, which are not susceptible to SARS-CoV-2 infection due to the ack of human ACE2, served as control. RNA was extracted from lung tissues were collected at day 4 and day 6 post infection and sequenced.

### Data mining

Data was accessed through NIH gene omnibus using accession numbers(GSE175996) (27) (28),(GSE171524), and (29) GSE147507. We also used the following platforms as described above: singlecell.broadinstitute.org or Skyline Immgen (www.immgen.org), data from Skyline Immgen was reported as expression values normalized by DEseq2. Further analysis of data was performed in GraphPad Prism.

### NR and anti-monomeric NAMPT antibody study

NR was added to standard mouse chow as previously described (71), with a target 800mg/kg/day. Anti-monomeric NAMPT antibody was obtained from LS-biosciences. Low dose was 0.4mg/kg, and high dose was 1.0mg/kg, administered intraperitoneally. The dose of SARS-CoV-2 used was 5 x 10^2 TCID50. Animals were weighed daily, and followed for 14 days post infection, at which they were euthanized. Plasma was collected after euthanasia and subject to metabolomic analysis.

### Metabolomics

The metabolomics were conducted as previously reported (60, 72). LC-MS analysis was performed on a ThermoFisher Scientific Q Exactive HF-X mass spectrometer equipped with a HESI II probe and coupled to a ThermoFisher Scientific Vanquish Horizon UHPLC system. Metabolites were extracted using 80% methanol and separated at 0.2 ml/min by HILIC chromatography at 45°C on a ZIC-pHILIC 2.1-mm i.d × 150 mm column (EMD Millipore) using 20 mM ammonium carbonate, 5 micromolar medronic acid, 0.1% ammonium hydroxide, pH 9.2, (solvent A) and acetonitrile (solvent B) with a gradient of: 0 min, 85% B; 2 min, 85% B; 17 min, 20% B; 17.1 min, 85% B; and 26 min, 85% B. Samples were analyzed using full MS scans with polarity switching for quantitation at: scan range 65 to 975 m/z; 120,000 resolution; automated gain control (AGC) target of 1E6; and maximum injection time (max IT) of 100 ms. A sample pool (QC) was produced by combining equal volume of each sample and analyzed using full MS scan at the beginning, middle and end of run sequence. MS/MS was also performed on the QC samples using separate runs for positive and negative mode analysis as follows: a full MS scan was acquired as described above, followed by MS/MS of the 10 most abundant ions at 15,000 resolution, AGC target of 5E4, max IT of 50 ms, isolation width of 1.0 m/z, and stepped collision energy of 20, 40 and 60. Metabolite identification and quantitation were performed using Compound Discoverer version 3.3SP1 (Thermo Fisher Scientific). Metabolites were identified from a mass list of verified compounds (high confidence annotations) and by searching the data against mzCloud database (www.mzcloud.org).

## Data availability statement

The original contributions presented in the study are included in the article/supplementary materials. Further inquiries can be directed to the corresponding author.

## Ethics statement

The animal study was reviewed and approved by Tulane University Institutional Animal Care And Use Committee.

## Author contributions

AI performed experiments and contributed to the writing of the manuscript. JM Performed analysis of some of the bioinformatic data and contributed to the writing of the manuscript. SS contributed to the data, bioinformatic, and biostatistic analysis and contributed to the writing of the manuscript. JK contributed to the Single Cell RNA Seq studies and contributed to the writing of the manuscript. XQ contributed to the performance of the animal studies in the SARS-COV-2 mouse model and contributed to the writing of the manuscript. JR contributed to the design and conception of the studies and and contributed to the writing of the manuscript. JGNG contributed to the conceptualization. Ma-M,CP, and ARG contributed to the metabolomics analysis. All authors contributed to the article and approved the submitted version.
